# Fixation of Acute Chondral Fractures in Adolescent Knees

**DOI:** 10.1177/1947603520941213

**Published:** 2020-07-16

**Authors:** Stian Kjennvold, Per-Henrik Randsborg, Rune B. Jakobsen, Asbjorn Aroen

**Affiliations:** 1 Orthopedics Department, Akershus University Hospital, Lørenskog, Norway; 2 Institute of Clinical Medicine, Campus Ahus, University of Oslo, Norway; 3 Department of Health Management and Health Economics, Institute of Health and Society, University of Oslo, Norway

**Keywords:** cartilage repair, repair, knee, joint involved, sports injury, diagnosis, arthroscopy, procedures

## Abstract

**Objectives:**

Chondral fractures are focal cartilage lesions without osseous attachment, most commonly seen in adolescent knees. They have limited capacity for intrinsic healing and traditional treatment has been removal of loose fragments. However, case reports of successful healing after fixation indicate that repair of the joint surface is possible. We wanted to evaluate the outcome in a cohort of patients who underwent fixation of acute chondral fractures in the knee.

**Design:**

Patients treated with fixation of a chondral fracture in the knee at our institution were invited to participate in a follow-up study. The mechanism of injury, fragment properties and complications were registered. Patients completed KOOS (Knee Injury and Osteoarthritis Outcome Score) and Lysholm questionnaires and performed a validated single leg hop test. Magnetic resonance imaging (MRI) was used to assess healing of the defect and the quality of the cartilage.

**Results:**

Ten patients with a median age at surgery of 15 years (12-17 years) and median follow-up of 5 years (2-9 years) were assessed. The lesions were located on the patella (*n* = 7), the trochlea (*n* = 2), and the lateral femoral condyle (*n* = 1). Median lesion size was 250 mm^2^ (1.9-6.0 cm^2^) All patients were treated within 2 months of injury (4-58 days). All patients returned to preinjury level of sports and MRI showed retained fragments that integrated well with surrounding cartilage at follow-up. Mean Lysholm score at follow-up was 90 (73-100).

**Conclusion:**

Fixation of traumatic chondral-only fragments using bioabsorbable implants may result in successful healing in adolescent patients and should be considered a treatment option in acute injuries.

## Introduction

Focal cartilage defects of the knee are common especially in the young and active population, and have been shown to impair quality of life similar to patients scheduled for knee replacement.^1-3^ Acute injuries can cause swelling, pain, and mechanical symptoms such as locking or instability. Focal cartilage lesions also predispose for early-onset osteoarthritis.^4,5^ Several surgical treatment options have been developed for chronic, symptomatic lesions but they all result in a mechanical inferior cartilage compared with the native hyaline cartilage. Controversy exists concerning the optimal treatment.^6,7^

While most acute knee cartilage injuries are osteochondral lesions, the structural anatomy of adolescent joint cartilage makes it susceptible to shear fractures without osseous attachment, a lesion known as a chondral fracture. Chondral fractures are often seen after a patellar dislocation, but they can also be caused by torsional forces or direct trauma to the knee. Since cartilage has no blood supply, it has been a widely accepted biological principle that once injured, cartilage does not heal. The absence of cancellous bone would suggest that pure chondral fractures have a very limited capacity for intrinsic healing. Traditional treatment of chondral fractures has therefore been removal of the loose fragments to prevent mechanical symptoms, followed by debridement or cartilage restoration procedures. However, case reports of successful healing after fixation of acute injuries indicate that repair of the native hyaline cartilage surface is possible (**Table 1**). This surgical technique is the only treatment that preserves the patient’s own hyaline cartilage, which in turn could vastly improve the prognosis for patients with acute isolated chondral trauma.

**Table 1.** Published Case Series on Fixation of Acute Chondral Fractures of the Knee.^8-16^

Authors	Fixation Method	No. of Cases	Patients with Follow-up MRI	Second-Look Arthroscopy	Time to Follow-up	Outcome
Maletius *et al*. (1994)	Fibrin sealant and polydioxanone pins	2	0	2	7 mo and 8 mo	Partial healing of defects
Nakamura *et al*. (2004)	Bioabsorbable pins	1	1	1	2 y 9 mo	Successful repair
Uchida *et al*. (2012)	Bioabsorbable pins	3	3	2	2 y	Successful repair
Chan *et al*. (2014)	Bioabsorbable suture anchors, absorbable suture, bone fixation nails	1	1	1	1 y	Successful repair
Nakayama *et al*. (2014)	Autograft bone pegs	1	1	1	2 y	Successful repair
Morris *et al*. (2016)	Poly-l-lactic acid chondral darts	1	1		1 y	Successful repair
Siparsky *et al*. (2017)	Chondral darts, sutures, and Tissel fibrin glue	3	2	2	18 mo median	Successful repair
Fabricant *et al*. (2018)	Combinations of bioabsorbable tacks, screws, suture, and fibrin glue	15	9	3	1 y median	Successful repair in 14/15
Churchill *et al*. (2019)	Combinations of bioabsorbable implants and metal screws	10	8	1	3 y median	Successful repair

The outcome of this surgical treatment is poorly established. The published case series are all quite small with limited time follow up. The case series have different definitions of successful repair, ranging from lack of clinical failure to healing of the lesion seen either on magnetic resonance imaging (MRI) or during second-look arthroscopy, or a combination of all methods.

Nevertheless, we have performed fixation of acute chondral fractures in a number of adolescent patients over the past decade. As this is a relatively new approach to the treatment of these injuries, we owe it to our patients and ourselves to critically and systematically evaluate the results in a scientific manner. Thus, the purpose of the current study was to determine clinical presentation, indication, limitations, and expected outcome of this treatment. The study hypothesis was that this retrospective investigation would demonstrate successful radiological healing with acceptable clinical results. Furthermore, we wanted to describe our surgical technique to standardize the procedure.

## Methods

This is a retrospective case series at a single institution. Inclusion criteria were patients who underwent fixation of an acute, isolated chondral fracture of the knee with bioabsorbable Meniscus Arrows (Con Med, Utica, NY) at our institution between 2008 and 2018. A chondral fracture was defined as absence of bone on the cartilage fragment assessed on MRI and perioperatively by the performing surgeon.

Surgery was performed if the lesion size and location indicated future symptoms for the patient and healing seemed possible with internal fixation as evaluated by one of the senior knee consultants. Repair was also performed in cases with a fragmented lesion if it was considered feasible. Fixation was not performed in lesions that that did not need repair due to small fragments or were considered irreparable due to excessive fragmentation. Lesions containing bone or with subchondral fractures were excluded.

A total of 13 patients with fixation of chondral fragments using Meniscus Arrows were identified from our medical records. All patients sustained a documented knee injury and had MRI preoperatively. Two patients were excluded as arthroscopy described cancellous bone attached to the loose fragments and were thus classified as osteochondral fractures. Eleven patients met the inclusion criteria. One patient was lost to follow-up, leaving data from 10 patients available for analysis.

Patients provided information about return to work, physical activity, and sport activities at follow-up. Complications or reoperations were registered. To assess clinical function, a standard knee examination was performed. Range of motion (ROM) was measured with a goniometer. The patient reported outcome was quantified by collecting the Knee Injury and Osteoarthritis Outcome Score (KOOS) and Lysholm score. To assess an objective clinical outcome, all patients were asked to perform a validated single leg hop test, which is a sequence of standardized jumps on injured and noninjured leg, respectively.^17^ The patients performed a series of 4 different distance jumps; a single leg hop, a triple hop, and a crossed triple hop, then a 6-m timed hop. All jumps were performed twice on each leg and the average of the 2 scores were calculated for each leg. To quantify the relative performance between the injured and uninjured leg, we used limb symmetry index (LSI), which is commonly used in rehabilitation of athletes to determine return to sports. LSI values of 90% or higher in all 4 jump categories was considered satisfactory.^18^

Postoperative MRI at follow-up (2-9 years), including a T2-mapping sequence was used to assess the healing of the defect and the quality of the cartilage. A priori sample size power calculation was not performed as no statistical hypothesis was to be confirmed or rejected. Continuous variables are presented as median values with spread, while categorical data are presented in percentage.

The study was approved by the Regional Ethical Committee of South Eastern Norway (REK 2015/2403). All patients provided a written informed consent to participate in the study.

## Results

Median age at surgery was 15 years (12-17 years) and the median follow-up time was 5 years (2-9 years). Eight of 10 patients were skeletally immature at the time of surgery. The injury mechanisms included patellar dislocation (*n* = 7), twisting of the knee (*n* = 2), and direct trauma (*n* = 1). The chondral fractures were located on patella (*n* = 7), trochlea (*n* = 2), and the lateral femoral condyle (*n* = 1). Median lesion size was 2.5 cm^2^ (1.9-6.0 cm^2^) based on arthroscopic measurements. One patient had fragmenting of the loose cartilage originating from a single lesion site. Median time to surgery was 27 days (4-58 days) (**Table 2**). None of the patients had other procedures performed at the time of index surgery apart from fixation of the loose fragment. One patient suffered a concomitant partial anterior cruciate ligament rupture and another patient had a complete medial collateral ligament injury. Both injuries were treated conservatively.

**Table 2.** Patient Demographics, Injury Properties, and Follow-up Time for All 10 Patients.

Gender	Age, years	Lesion Size (mm)	No of Arrows	Location	Trauma	Time to surgery, days	Follow-up, years
Female	15	16 × 12	5	Patella	Patellar dislocation	20	8
Female	16	16 × 12	7	Patella	Patellar dislocation	12	5
Male	15	20 × 15	4	Patella	Patellar dislocation	14	7
Male	14	23 × 18	5	Trochlea	Torsional trauma	13	5
Female	12	10 × 20	3	Patella	Patellar dislocation	58	5
Male	13	23 × 26	14	Trochlea	Direct trauma	4	4
Female	15	20 × 15	6	Patella	Patellar dislocation	34	3
Female	17	10 × 20	2	Patella	Patellar dislocation	47	2
Male	15	25 × 15 + 10 × 10	10	Lateral femoral condyle	Patellar dislocation	14	9
Male	13	20 × 10	4	Trochlea	Torsional trauma	47	3

Seven of the 10 patients were competing in national or international level sports at the time of injury (2 soccer players, 1 handball player, 1 hockey player, 2 gymnasts, and 1 basketball player). The last 3 patients sustained their injuries during recreational sport activities.

**Figure 1.**
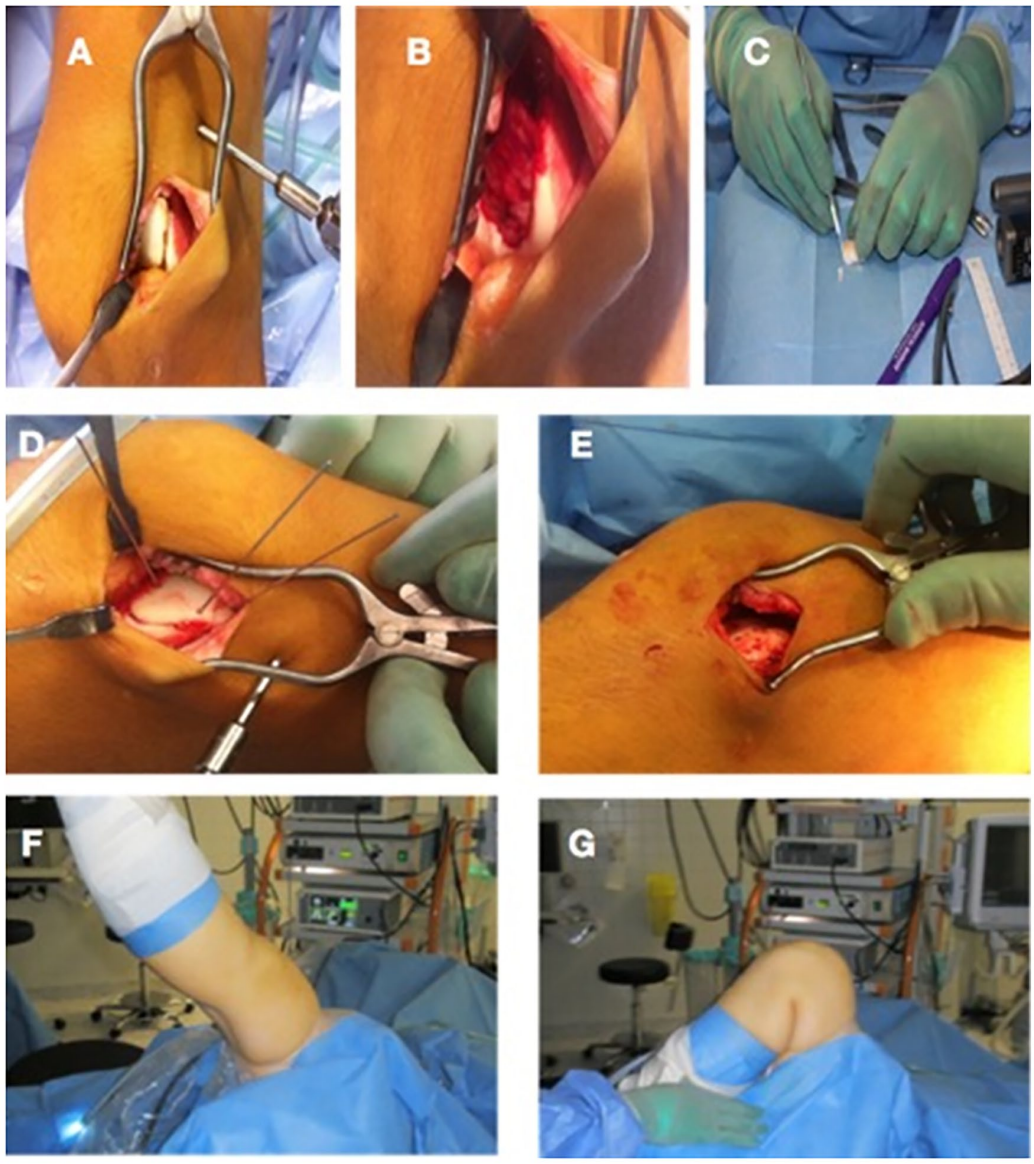
Operative technique. The lesion is reached through a mini-arthrotomy (**A**). If completely loose, the lesion is removed from the joint (**B**). If swelling has occurred the cartilage has to be sized to fit the lesion (**C**). Temporary fixation is obtained with Kirschner wires (**D**) before final fixation with Meniscus Arrows (**E**). The joint is taken through full range of motion to ensure stability (**F** and **G**).

**Figure 2.**
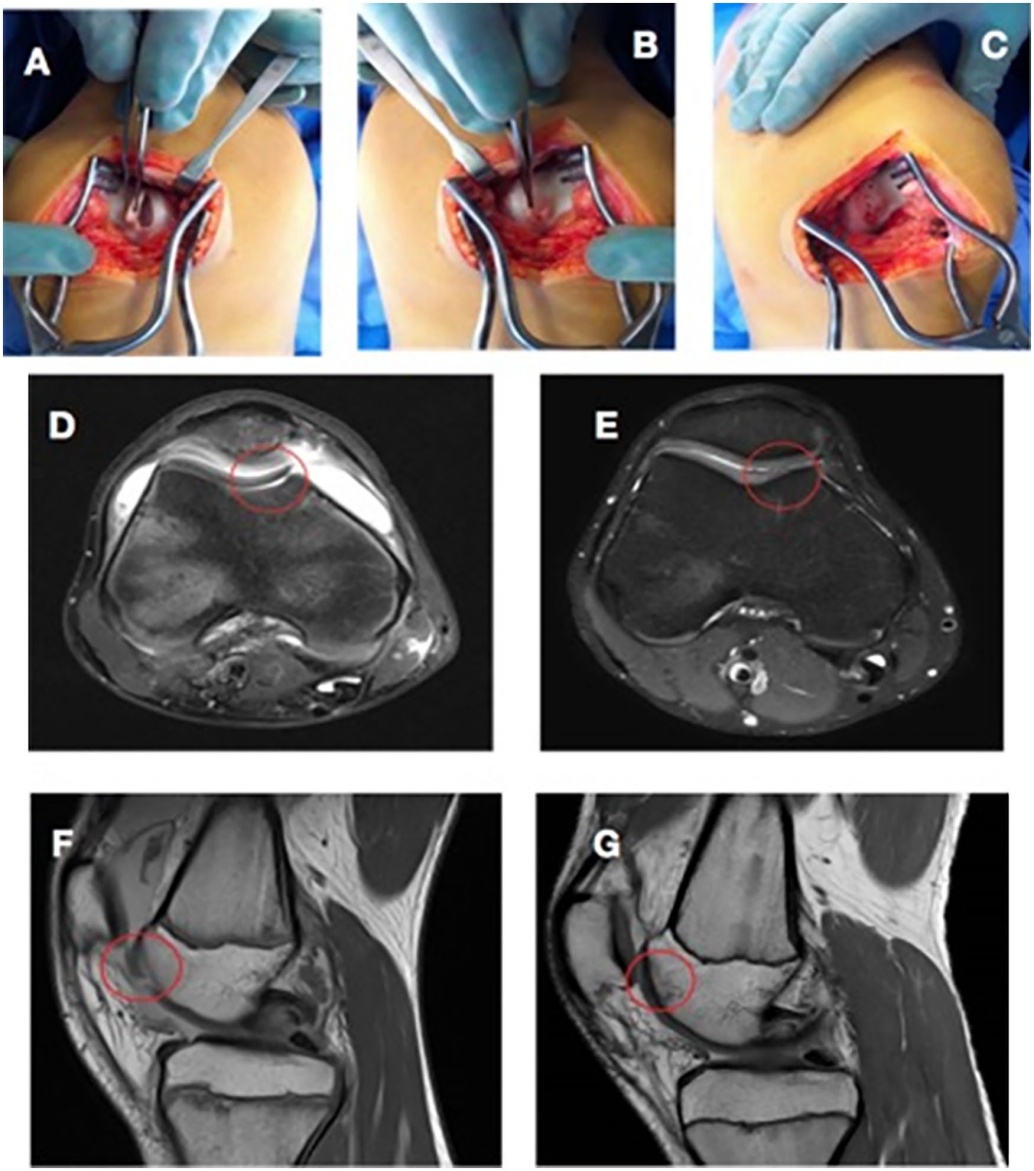
A 14-year-old male patient who sustained a trochlear chondral fracture while playing soccer (**A**, **D**, and **F**). The lesion was hinged on the lateral side and was not detached (**B**). The lesion bed was debrided and subchondral bone was drilled before fixation with 5 meniscal arrows (**C**). Magnetic resonance imaging 3 years postoperatively (**E** and **G**) shows healing of the cartilage lesion and partial regression of the subchondral changes.

All patients were treated with the same surgical technique using bioabsorbable Meniscus Arrows for fixation (**Fig. 1**). A mini-arthrotomy following diagnostic arthroscopy was performed in all patients. Although it is possible to perform fixation arthroscopically, a mini-arthrotomy reduces the risk of cleavage of the chondral fragment. The loose chondral fragments were taken out and shaped to correct size if swelling had occurred. If hinged, the chondral fragment was not detached (**Fig. 2**). The lesion bed was prepared with gentle debridement of remaining cartilage fragments or scar tissue and subsequent subchondral drilling. The fragment was then temporarily fixed using Kirschner wires, before final fixation with Meniscus Arrows. Adequate fixation of the fragment was tested by taking the knee through full range of motion several times, making sure there were no mechanical symptoms. Then careful irrigation was performed before wound closure.

Postoperatively, the patients were allowed immediate full range of motion but strictly weight bearing on a straight leg only for 6 weeks. They were followed by external physiotherapists with general guidelines for postoperative rehabilitation. They were recommended to stay away from any sports for a minimum of 3 months and informed of up to a year of rehabilitation before full return to pivoting sports. Patients with patellar dislocation as their index injury were advised to use a patella stabilizing brace for the first 3 months when returning to pivoting activities.

**Figure 3.**
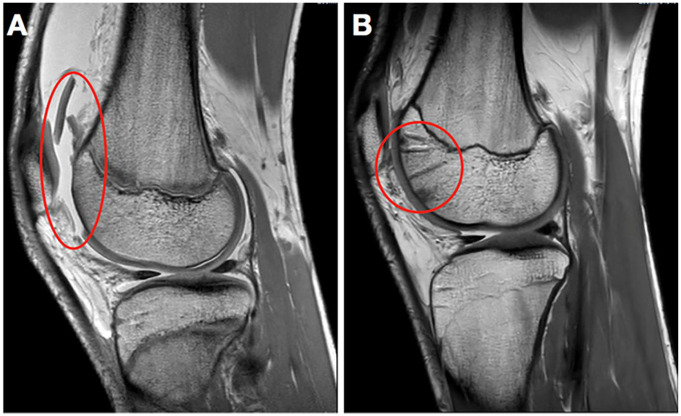
A 13-year-old male patient with a trochlear chondral lesion in his right knee caused by direct trauma while playing football (**A**). Magnetic resonance imaging shows healing of the chondral fracture and good integration with surrounding cartilage after 1 year (**B**).

**Figure 4.**
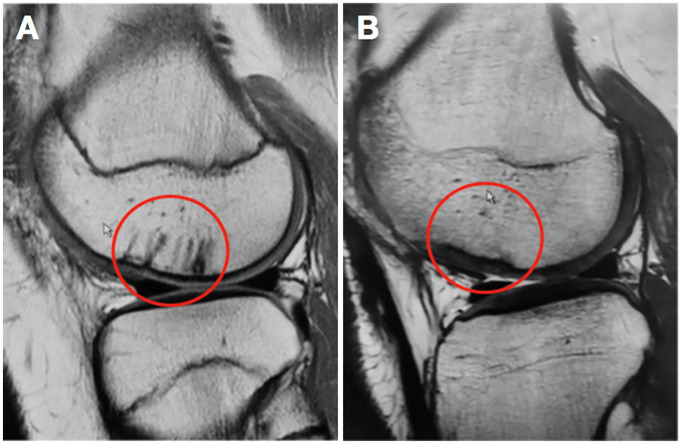
A 15-year-old male patient with fixation of a multifragmented chondral lesion on the lateral femoral condyle demonstrating postoperative subchondral changes from the drilling (**A**) Magnetic resonance imaging 2 years after surgery shows regression of the postoperative subchondral changes (**B**).

At follow-up after 2 to 9 years, all 10 patients had MRI scans with T2-mapping to assess the healing of the fragment and the quality of the cartilage. We found that all 10 lesions were intact at the time of follow up. Some showed slight signs of thinning or thickening, but they all seemed to heal and integrate well with underlying bone and surrounding cartilage. All knees also showed partial or full regression of the subchondral changes caused by preparation of the lesion site (**Figs. 3** and **4** )

**Figure 5.**
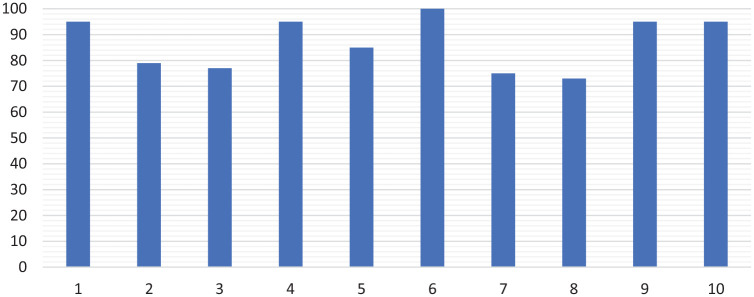
Postoperative Lysholm knee scores for all 10 patients.

The median Lysholm score was 90 (73-100). Five patients scored 95 or more (**Fig. 5**).

**Figure 6.**
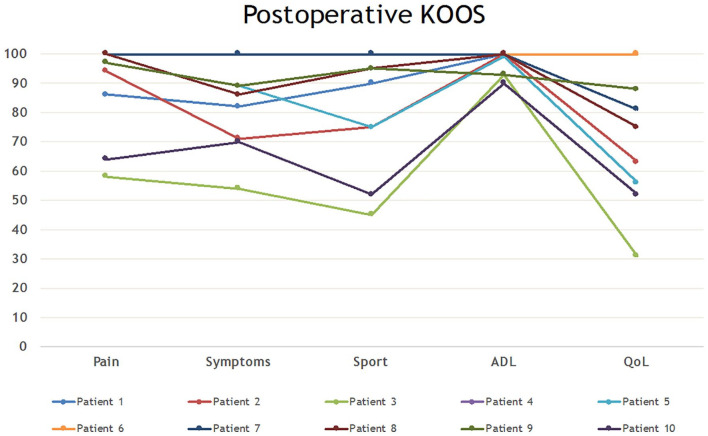
KOOS (Knee Injury and Osteoarthritis Outcome Score) scores for all 10 patients in all subscales.

Three patients scored 100 for all subscales of the KOOS (**Fig. 6**). Out of 10 patients, 7 scored from 80 to 100 for all subscales except QOL (Quality of Life). The 2 lowest scoring patients on all subscales are patients not actively involved in sports pre- or postoperatively.

One patient was unable to conduct the single leg hop test on both the injured and uninjured leg. MRI showed healing of the defect and clinical examination revealed full ROM, ligamentous stability and no mechanical symptoms. The patient did not report any pain from the knee. For the remaining 9 patients LSI was well above 90% for all 4 jump categories (**Table 3**).

**Table 3.** Results of Single Leg Hop Test with Limb Symmetry Index (LSI) at Follow-up.

Single Leg Hop Test	Uninjured Leg	Injured Leg	LSI (%)
Single hop, cm	141 (107-191)	127 (87-191)	91.2
Triple hop, cm	459 (305-616)	450 (299-569)	94.4
Crossover hop, cm	392 (210-520)	374 (199-559)	96.1
6-m hop, s	1.97 (1.74-3.17)	2.01 (1.72-3.13)	98.0

All patients involved in sports returned to their preoperative level after surgery at a median of 9 months (7-11 months). We had no registered complications. Two patients had additional surgery to their knee at a later stage. One patient had arthroscopic removal of loose cartilage fragments after suffering a new multifragmented cartilage injury 3 years postoperatively. The originally fixed lesion viewed arthroscopically had healed. One patient had a medial patellofemoral ligament reconstruction and tibial tuberosity transfer surgery because of recurrent patellar instability. The index chondral lesion was evaluated as healed during surgery.

## Discussion

The main finding in this study is that fixation of chondral fractures with bio-absorbable Meniscus Arrows yields good midterm clinical and radiological results. There were no severe complications and the chondral fragments integrated nicely onto bone and with surrounding cartilage in all 10 patients. All patients involved in sports preoperatively returned to the same level of sport after surgery at a median of 9 months. Previous dogma that cartilage lesions do not heal once injured do not seem to be the case in adolescent knees with an acute, traumatic chondral fracture.

Only 1 other study has reported midterm results following fixation of chondral fractures. Churchill *et al*.^16^ published a case-series of ten patients with a median follow-up of 3 years. Like us, they found no failures and reported excellent outcome at follow-up. Unlike our study, the patients had been treated with a variety of implants, including metal screws and not all patients had a postoperative MRI.^16^

Our study confirms the findings of previous case reports (**Table 1**) and expands on existing literature with 5-year median follow-up, clinical, radiological, and patient-reported outcome measures. It lends support to the increasing trend of surgical fixation of acute chondral fractures. The chondral lesions in this cohort were all quite large and located in the contact surface of the tibiofemoral or patellofemoral joint, and surgery was therefore indicated. However, we do recognize that we do not fully know the conservative treatment outcome of these injuries. Further randomized controlled trials would be necessary to confirm the effectiveness of the procedure.

We have presented a standardized operation technique and all patients have been operated in the same manner. The surgical method we describe is relatively easy to perform and is the only known method that preserves the native hyaline cartilage of the patients. If it fails, we still have other procedures such as cell-based therapies, mosaicplasty, or allograft available.

In our study, 7 out of 10 chondral fractures were caused by a patellar dislocation. We know that cartilage injuries are common after first time dislocations and routine MRI should therefore be considered in patients with significant hemarthrosis.^19^ Most traumatic, first time patellar dislocations can be treated conservatively.^20^ However, it is important that these patients are carefully evaluated for risk factors for recurrent patella instability to avoid new dislocations and potential re-injury to the treated chondral fracture. The risk for recurrent patellar instability increases with patella alta, increased tibial tubercle to trochlear groove distance and trochlear dysplasia.^21^ The risk for recurrent instability is up to 80% if 2 or more risk factors are present but can be as low as 10% in traumatic dislocations without associated risk factors.^22^ This is in line with our cohort where 1 out of 7 patients had recurrent patellar instability to such an extent that stabilizing surgery was indicated. Fixation of chondral injuries will restore part of the mechanical stability in the patellofemoral joint and might reduce the risk of future dislocations, but further studies are needed to confirm this.

We recognize that our study has several limitations, such as the limited number of patients and retrospective study design. Furthermore, preoperative patient-reported outcome measures were not collected, which prevented quantification of the improvement after surgery. A majority of patients in this case series were active athletes that might have higher base line scores than the average adolescent population. However, the LSI was well above 90% for all four jump categories which is comparable to the level of healthy participants in other studies.^18^

We did not see any failures of the fixation which could indicate that this is not regularly occurring with this treatment modality. The results might be different in older age groups or in combination with other knee injuries (such as ligament injuries). Larger prospective studies would be needed to establish this.

The present case series is one of the largest to date and adds to existing literature with a median follow-up time of 5 years. The results suggest that fixation of a chondral fracture is the only treatment of a focal cartilage injuries that fully preserves the native hyaline cartilage and a modality that should be considered by orthopedic surgeons treating these patients.

## Conclusion

Despite the limited healing potential of cartilage lesions without cancellous bone the results of the current study suggest that restoration of the joint surface can be obtained in acute injuries. The study shows radiological healing in all patients and excellent clinical results in some. Fixation of traumatic chondral-only fragments using bioabsorbable implants may result in successful healing in adolescent patients and should therefore be considered a treatment option in acute injuries.
